# Antiplatelet Prophylaxis Reduces the Risk of Early Hepatic Artery Thrombosis Following Liver Transplantation in High-Risk Patients

**DOI:** 10.3389/ti.2024.13440

**Published:** 2024-12-18

**Authors:** Iulia Minciuna, Jeroen De Jonge, Caroline Den Hoed, Raoel Maan, Wojciech G. Polak, Robert J. Porte, Harry L. A. Janssen, Bogdan Procopet, Sarwa Darwish Murad

**Affiliations:** ^1^ Erasmus Medical Center Transplant Institute, Erasmus University Medical Center Rotterdam, Rotterdam, Netherlands; ^2^ University of Medicine and Pharmacy Iuliu Hatieganu, Cluj-Napoca, Romania; ^3^ Octavian Fodor Gastroenterology Institute, Iuliu Hațieganu University of Medicine and Pharmacy, Cluj-Napoca, Romania; ^4^ Department of Surgery, Division of Hepato-Pancreato-Biliary and Transplant Surgery, Erasmus Transplant Institute University Medical Center Rotterdam, Rotterdam, Netherlands; ^5^ Toronto General Hospital, Toronto, ON, Canada

**Keywords:** liver transplantation, hepatic artery thrombosis, risk factors, antiplatelet prophylaxis, 1-year graft survival, hepatic artery thrombosis

## Abstract

The prevention of hepatic artery thrombosis (HAT) is pivotal for graft survival immediately after liver transplantation (LT). This study aimed to identify risk factors (RF) for early HAT (eHAT) and assess the benefit of antiplatelet prophylaxis (AP). This retrospective single-center study included 836 adult patients who underwent LT between 2007 and 2022. AP was administered for 3 months in N = 127 patients for surgical reasons. In total, 836 patients underwent LT, of whom 5.5% developed eHAT. In multivariable analysis, arterial anastomotic redo (aHR = 4.33), arterial reconstruction (aHR = 3.72) and cryptogenic liver cirrhosis (aHR = 4.25) were independent RFs for eHAT and AP appeared to be protective (aHR = 0.18). Indeed, in patients with at least one RF who received AP (RF+AP+, n = 94), the eHAT rate was significantly lower (3.2% vs. 21.3%, *p* < 0.001) than in those with RF who did not receive AP (RF+AP−, n = 89). The effect was even more pronounced when focusing on surgical RF alone (i.e., redo and/or reconstruction) with an additional improvement in 1 year graft survival of 85.3% vs. 70.4%, *p* = 0.02. AP did not pose an increased risk of bleeding. In conclusion, the main RFs for eHAT include arterial anastomotic redo, arterial reconstruction and cryptogenic liver cirrhosis as LT indications. Our results suggest that AP may protect against eHAT development in these high-risk patients.

## Graphical Abstract

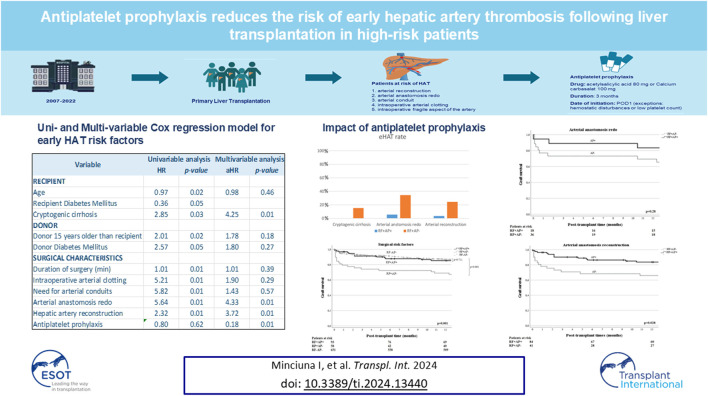

## Introduction

Despite advancements in surgical technique, postoperative care, and immunosuppression, liver transplantation (LT) continues to be associated with morbidity and mortality, particularly in the early postoperative period [1].

The most feared complications are vascular in nature and can lead to graft dysfunction, graft loss or even recipient death [2]. With a reported incidence of 4.4%–9%, hepatic artery thrombosis (HAT) is a severe complication that can result in liver necrosis, abscess formation, ischemic biliopathy and graft failure requiring re-transplantation in up to 50% of cases [3, 4]. Depending on the time of occurrence, HAT can be subdivided as early (i.e., within 2 months) and late (i.e., beyond 2 months post-LT) [3]. At the core of this division are the differences in terms of risk factors (RFs), clinical presentation, treatment options and potential outcomes.

Therefore, prevention, early detection and timely management of early HAT (eHAT) are of paramount importance for graft and patient survival, especially in patients at high risk of developing HAT. Many transplant teams, including ours [5] have implemented close surveillance of all vascular anastomoses with color Doppler to provide early detection in the immediate post-operative period, and facilitate timely intervention. Moreover, it is known that during transplant, constant platelet activation and aggregation result in thromboxane development and fibrinogen activation, which subsequently predispose to arterial thrombosis and ischemia/reperfusion injury [6]. Prevention of eHAT by antiplatelets (i.e., antiplatelet prophylaxis, AP) has therefore received considerable attention. However, there is unfortunately considerable heterogeneity in the reported studies, the majority of which are retrospective and all of which are observational in nature, in terms of study populations (adults, children, living or deceased donors), in- and exclusion criteria, reported outcomes (early HAT, late HAT, any HAT), type of antiplatelet therapy and duration of therapy. In fact, only 4 studies [7–10] have evaluated the effect of AP on the development of HAT in adult deceased donor liver transplant populations, of which only 1 [9] assessed the effect of early HAT and the remainder on HAT at any time point. Based on these, and other studies (in pediatric or living donor populations), the most cited review from the ILTS group ERAS4OLT recently recommended antiplatelet prophylaxis in all liver transplant patients [11]. However, largely due to the same heterogeneity, the group judged their recommendation as low-quality evidence and the effect size as small. In addition, a recent multicenter study by Oberkofler et al. showed that the benefits of antiplatelets may extend beyond thromboprophylaxis, as the authors observed a reduction in acute cellular rejection rates [12]. However, in this multicenter study, only 4 centers used aspirin routinely in all patients, while the other 13 administered AP only at the surgeon’s discretion. It is therefore still unclear whether AP is beneficial for all patients or only for those with a high risk of HAT. Moreover, there are some concerns regarding the risk of bleeding with the use of antiplatelets, in particular in the early postoperative period [13].

Therefore, the **aim** of this study was to identify risk factors for the development of eHAT and assess whether and in whom antiplatelet prophylaxis (AP) reduces the risk of eHAT.

## Materials and Methods

### Study Population

All patients aged 18 years or older who underwent LT at our center between January 2007 and September 2022 were included in the study. Exclusion criteria included re-transplantation, combined organ transplantation, and chronic use of antiplatelets for non-liver-related (cardiovascular) reasons. As we were interested in early HAT only, patients who developed late HAT (i.e., after 2 months) were also excluded.

Antiplatelet prophylaxis (AP, i.e., acetylsalicylic acid 80 mg or carbasalate calcium 100 mg) was administered for 3 months to patients for any of the following surgical reasons: 1) need for arterial reconstruction (defined as any additional arterial anastomosis between the donor and recipient hepatic arteries in the case of an anatomical variant), 2) arterial anastomosis redo (defined as immediate remaking of the arterial anastomosis in the case of suboptimal arterial inflow during the transplantation), 3) arterial conduit, 4) intraoperative arterial thrombus formation developed during implantation prompting immediate thrombectomy, or 5) a fragile aspect of the artery [e.g., due to previous transarterial radio- (TARE) or chemo-embolization (TACE) or atherosclerosis]. The arterial anastomosis was kept as short as possible and performed in an end-to-end manner to prevent kinking while considering the diameters of both the donor and recipient arteries. The most frequent site of anastomosis was at the level of the recipient’s proper hepatic artery, just above the gastroduodenal artery.

Arterial flow was assessed intra-operatively by *in situ* Doppler Ultrasound, placing the probe directly on the hepatic artery. Immediately after abdominal closure [referred to as postoperative day (POD) 0], as well as on POD1 and POD7, arterial flow was assessed routinely by Doppler ultrasound performed by transplant hepatologists with extensive ultrasonography experience. This was followed by a contrast‐enhanced computed tomography (CT) scan if the results suggested the presence of a vascular complication within the graft. After discharge, all patients remained life-long in follow-up at our center.

### Data Collection

Data were collected retrospectively from electronic patient records. The primary endpoint was early HAT (eHAT), defined as a thrombotic occlusion of the hepatic artery, resulting in the absence of a hepatic arterial signal at the hilum or in the intrahepatic arterial branches on Doppler Ultrasound and/or a non-enhancing filling defect on contrast-enhanced CT scan, occurring within 2 months after LT. Secondary outcomes included graft and recipient survival. Patients were followed from the time of transplant until re-transplantation (i.e., graft failure), death (i.e., recipient mortality) or last follow-up (September 2022). Graft survival was calculated from the time of transplantation until re-transplantation or death, with censoring at the time of the last follow-up. Patient survival was calculated from the time of transplantation until death or last follow-up, irrespective of re-transplantation.

The following recipient variables were collected at the time of LT: age, gender, BMI, ethnicity, blood group, transplant indication, MELD score, type of graft [i.e., donation after brain death (DBD), donation after circulatory death (DCD) or living donor liver transplantation (LDLT)], metabolic co-morbidities (i.e., hypertension, Type II diabetes mellitus, obesity, dyslipidemia), prothrombotic condition (protein C or S deficiency, JAK2 mutation, Factor V Leiden mutation, antiphospholipid syndrome, antithrombin III deficiency), history of pre-LT vascular interventions (TACE, TARE), CMV and EBV mismatch status. The following donor characteristics were collected: age, gender, BMI, Donor Risk Index (DRI) [9], diabetes mellitus and smoking status. The collected data at the time of surgery included cold ischemia time, warm ischemia time, duration of surgery, arterial reconstruction, need for arterial anastomosis redo, use of arterial conduit, intraoperative arterial thrombus formation, use of *ex-situ* machine perfusion or normothermic regional perfusion, blood-loss volume, use of perioperative blood products and the percent of graft steatosis. Other variables collected post-LT included total duration of hospitalization, hemorrhagic events during the first 3 months following LT, need for re-transplantation, and 1-year graft and patient survival.

### Statistical Analysis

The primary outcome was the development of eHAT. Secondary outcomes were graft and patient survival, calculated by the Kaplan-Meier method. Quantitative variables were expressed as medians with extreme values (range) and compared using Student’s t-test or Wilcoxon test as appropriate. Qualitative variables were expressed as numbers and percentages and compared using Chi-square or Fisher’s exact tests, as appropriate. Patients who developed eHAT and those who did not were compared with regard to recipient, donor and surgical factors.

Risk factors (RFs) for the development of eHAT were detected by first performing univariable Cox regression analyses on all variables of interest, taking into account the time to eHAT. Subsequently, factors that were statistically significant (*p* < 0.05) in the univariable analysis were considered for inclusion in a multivariate COX regression analysis to identify independent predictors of eHAT. As we were interested in the effect of antiplatelet prophylaxis (the variable of primary interest), we decided to add this variable to the multivariable model, regardless of the univariate results. As we predicted that we would run into the risk of overfitting in the multivariable model due to the small number of events and many potential risk factors, we decided to go for a multivariable model with the best fit, as defined as the smallest AIC (Akaike Information Criterion), and the highest Area Under Receiver Operating Characteristic (AUROC). Variable selection was done by back-step, forward-step and manual methods, to keep all options open and find the one model with the best fit.

Finally, within the group of patients with at least one of the identified independent RFs (i.e., RF+), we compared eHAT development in those who received AP (RF+AP+) to those who did not (RF+AP−), based on the Chi-square test. Similarly, 1-year graft and patient survival were compared using the log-rank test.

All statistical analyses were performed using commercially available statistical software (SPSS Inc., Chicago, IL). A *p*-value of <0.05 was considered statistically significant.

## Results

In total, 839 patients who underwent primary liver-only LT were initially included in the study. For the purposes of our study, we decided to exclude 3 patients who developed HAT within hours after liver transplantation, given the fact that they did not have a chance of being exposed to AP, even if indicated, thus leading to a final number of 836 patients included in the analysis. These were followed for a median time of 48.6 months (range 0.02–189.84). Overall, 1 year graft survival was 85.7% (95% CI: 84.5–86.9) and patient survival was 90% (95% CI: 88.9–91.1).

Recipient characteristics, donor characteristics and surgical details of the total population are presented in detail in Table 1. Briefly, patients were an average of 54 (18–72) years old, and were mainly men (62.6%) with HCC (32.1%) and cholestatic liver disease (24.3%) being the main indications. The median MELD was 22 (6–40) and 63% received a graft from a DBD and 32.8% from a DCD donor.

**TABLE 1:** Baseline characteristics of 836 patients undergoing primary liver transplantation at our institution between 2007 and 2022.

Variables	Overall n = 836	Early HAT n = 46	No HAT n = 790	*p*-value
Recipient characteristics			
Age (years)	54 (18–72)	50 (19–70)	55 (18–72)	0.23
Recipient sex (male)	523 (62.6%)	32 (69.6%)	491 (62.2%)	0.31
Ethnicity			
Caucasian	575 (68.8%)	36 (78.3%)	539 (68.2%)	0.15
Asian	27 (3.2%)	0	27 (3.4%)	0.20
Black	40 (4.8%)	3 (6.5%)	37 (4.7%)	0.92
Other	85 (10.2%)	4 (8.7%)	81 (10.3%)	0.73
BMI (kg/m^2^)	25.5 (15.4–46.8)	25.9 (19.4–39.8)	25.4 (15.4–46.8)	0.30
Blood type			
O	346 (41.4%)	19 (41.3%)	327 (41.4%)	0.99
A	326 (39.0%)	18 (39.1%)	308 (39.0%)	0.98
B	109 (13.0%)	6 (13.0%)	103 (13.0%)	0.99
AB	55 (6.6%)	3 (6.5%)	52 (6.6%)	0.98
Liver disease etiology			
Viral	143 (17.1%)	6 (13.0%)	137 (17.3%)	0.45
ALD	137 (16.4%)	5 (10.9%)	132 (16.7%)	0.29
MASH	69 (8.3%)	3 (6.5%)	66 (8.4%)	0.66
PBC/PSC	203 (24.3%)	9 (19.6%)	194 (24.6%)	0.44
AIH	24 (2.9%)	1 (2.2%)	23 (2.9%)	0.77
Acute liver failure	76 (9.1%)	4 (8.7%)	72 (9.1%)	0.92
Metabolic	39 (4.7%)	2 (4.3%)	37 (4.7%)	0.91
Vascular	6 (0.7%)	0	76 (0.8%)	0.55
Cryptogenic	35 (4.2%)	5 (10%)	30 (3.8%)	**0.02**
HCC	268 (32.1%)	14 (30.4%)	254 (32.2%)	0.80
Pre-LT TACE/TARE	112 (13.4%)	4 (8.7%)	108 (13.7%)	0.33
MELD Score	22 (6–40)	24 (8–40)	22 (6–40)	0.43
Prothrombotic RF	10 (1.2%)	0	10 (1.3%)	0.44
Hypertension	131 (15.7%)	6 (13.0%)	125 (15.8%)	0.61
Diabetes Mellitus	174 (20.8%)	4 (8.7%)	170 (21.5%)	**0.04**
Obesity	39 (4.7%)	2 (4.3%)	37 (4.7%)	0.91
Dyslipidemia	17 (2%)	1 (2.2%)	16 (2%)	0.94
CMV mismatch	144 (17.2%)	5 (10.9%)	139 (17.6%)	0.24
EBV mismatch	32 (3.8%)	1 (2.2%)	31 (3.9%)	0.54
Donor characteristics			
Age (years)	53 (7–88)	51 (8–78)	53 (7–88)	0.52
Sex (male)	425 (50.8%)	19 (41.3%)	406 (51.4%)	0.18
BMI (kg/m^2^)	25 (10–42)	25 (19–35)	25 (10–42)	0.68
Donor Risk Index (DRI)	1.8 (0.9–3.3)	1.85 (0.9–2.5)	1.84 (0.9–3.3)	0.79
Diabetes mellitus	39 (5.3%)	5 (12.2%)	34 (4.9%)	**0.04**
Smoking	398 (47.7%)	22 (47.8%)	376 (47.7%)	0.70
Graft steatosis	309 (39.2%)	17 (40.5%)	292 (39.1%)	0.86
Donor 10 years older	231 (28.4%)	17 (37%)	214 (27.9%)	0.18
Donor 15 years older	178 (21.3%)	16 (34.8%)	162 (20.5%)	0.02
Type of graft			
DBD	527 (63.0%)	29 (63.0%)	498 (63.0%)	0.99
DCD	274 (32.8%)	14 (30.4%)	260 (32.9%)	0.72
Living Donor	34 (4.1%)	3 (6.5%)	31 (3.9%)	0.38
Domino	1 (0.1%)	0	1 (0.1%)	0.80
Surgical characteristics			
Surgery duration (min)	358 (154–760)	389 (234–570)	357 (154–760)	**0.03**
Machine perfusion	105 (12.6%)	3 (6.5%)	102 (12.9%)	0.20
DHOPE	77 (9.2%)	5 (10.8%)	74 (9.3%)	0.88
NRP	24 (2.8%)	0	24 (3.0%)	0.21
NMP	4 (0.5%)	0	4 (0.5%)	0.61
Blood loss (L)	3.5 (0.3–58)	3.9 (0.3–20)	3.5 (0.4–58)	**0.04**
Cold ischemia (min)	362 (109–1,031)	373 (124–759)	362 (109–1,031)	0.32
Warm ischemia (min)	28 (14–80)	28 (14–57)	28 (14–80)	0.95
RBC transfusion	620 (74.2%)	34 (73.9%)	586 (74.2%)	0.96
RBC units	4 (1–48)	4.5 (1–20)	4 (1–48)	0.94
FFP use	587 (70.2%)	33 (71.7%)	554 (70.1%)	0.81
FFP units	6 (1–56)	6 (1–25)	6 (1–56)	0.68
Plt transfusion	404 (48.3%)	17 (37.0%)	387 (49.0%)	0.11
Plt units	2 (1–11)	2 (1–4)	2 (1–11)	0.64
Fibrinogen use	403 (48.2%)	16 (34.8%)	387 (49.0%)	0.06
Tranexamic acid	610 (73.0%)	37 (80.4%)	573 (72.5%)	0.24
Prothrombin complex	120 (14.4%)	6 (13.0%)	114 (14.4%)	0.79
Intraoperative arterial	27 (3.2%)	6 (13.0%)	21 (2.7%)	**0.01**
Thrombus formation			
Arterial conduit	15 (1.8%)	4 (8.7%)	11 (1.4%)	**0.01**
Supraceliac conduit	11 (1.3%)	2 (4.3%)	9 (1.3%)	0.06
Infrarenal conduit	4 (0.5%)	2 (4.3%)	2 (0.3%)	**0.01**
Arterial redo	44 (5.3%)	10 (21.7%)	34 (4.3%)	**0.01**
HA reconstruction	125 (15.0%)	13 (28.3%)	112 (14.2%)	**0.01**
Peri-anastomotic bile leak^a^	23 (2.8%)	3 (6.5%)	20 (2.5%)	0.11

Results are expressed as N (%) or median (range). Variables were compared between patients who developed eHAT (n = 46) and those who did not (n = 790).

(e)HAT, (early) hepatic artery thrombosis; LT, liver transplantation; BMI, body mass index; ALD, alcohol-related liver disease; MASH, metabolic dysfunction associated steatohepatitis; PBC, primary biliary cholangitis; PSC, primary sclerosing cholangitis; AIH, auto-immune hepatitis; HCC, hepatocellular carcinoma; TACE, trans arterial chemoembolization; TARE, trans arterial radioembolization; MELD, Model for end-stage liver disease; RF, risk factor; CMV, cytomegalovirus; EBV, Epstein-Barr virus; DBD, donation after brain death; DCD, donation after cardiac death; DHOPE, dual hypothermic oxygenated machine perfusion; NRP, normothermic regional perfusion; NMP, normothermic machine perfusion; RBC, red blood cells; FFP, fresh frozen.

^a^ Bile leak preceding HAT.

The bold values indicate statistical significance.

In total, 127 (15.2%) patients received AP for 3 months, for the following reasons: arterial reconstruction (n = 84, 66.1%), and/or arterial anastomosis redo (n = 18, 14.2%), and/or arterial conduit (n = 5, 3.9%), and/or thrombectomy of intraoperatively formed arterial thrombus (n = 13; 10.2%) or fragility of the artery (n = 7, 5.5%). The majority of patients (55.9%) had a combination of the above. In total, 90.6% of these patients started AP on POD 0–5 (range: POD 0–18), with the exception of n = 12 subjects who were delayed to POD 7–18 due to fear of bleeding. In addition, all patients received high-dose prophylactic LWMH (i.e., nadroparin 5700 IU) during the ICU stay and normal dose (i.e., nadroparin 2850 IU) on admission. Patients receiving AP had significantly lower intraoperative blood loss (median 2,800 vs. 3,500 mL, *p* = 0.03), higher DRI (median 1.96 vs. 1.81, *p* < 0.05) but similar postoperative coagulation parameters such as median INR (2.0 vs. 1.9, *p* = 0.49), factor V (0.27 vs. 0.27, *p* = 0.68), antithrombin (0.38 vs. 0.39, *p* = 0.89) and platelet count (101.7 × 109 vs. 97 × 109, *p* = 0.52) than those who did not receive AP. Moreover, the use of AP was not associated with increased hemorrhagic events in the first 3 months post-LT (10.2% vs. 9.6%, *p* = 0.82).

### Characteristics of the Population That Developed Early HAT

In the total population, 46 (5.5%) patients developed eHAT. The median time to diagnosis was 4 days (range 0–50) and 71.7% of HAT occurred within the first week.

Patients who developed eHAT were more likely to have cryptogenic cirrhosis (10.0% vs. 3.8%, *p* = 0.02) but less likely to have pre-LT diabetes mellitus (8.7% vs. 21.5%, *p* = 0.04), than those without eHAT (Table 1). Moreover, patients who developed eHAT were significantly more likely to undergo hepatic artery reconstruction (28.3% vs. 14.2%, *p* < 0.01), arterial anastomosis redo (21.7% vs. 4.3%, *p* < 0.01), arterial conduit placement (8.7% vs. 1.4%, *p* < 0.01), or thrombectomy of an intra-operatively formed arterial clot (13% vs. 2.7%, *p* < 0.01). Similarly, the overall duration of surgery was significantly longer (389 vs. 357 min, *p* = 0.03) and patients had more intraoperative blood loss (3,912 vs. 2,500 mL, *p* = 0.04). As for donor factors, patients with eHAT were significantly more likely to receive a graft from a diabetic donor (12.8% vs. 5.2%, *p* = 0.04). Although both recipient and donor ages were not significantly different between the groups, we also evaluated the impact of an age difference between donor and recipient in 5-year increments. While a difference of 5 or 10 years was not significant for either older donors (*p* = 0.76 and *p* = 0.14, respectively) or recipients (*p* = 0.64 and *p* = 0.33, respectively), a difference in age with a donor 15 years older than the recipient was significantly more common in patients with eHAT compared to those without (34.8% vs. 20.5%, *p* = 0.02). The reverse situation (i.e., recipient 15 years older) was not found to be associated with eHAT development (*p* = 0.51).

Interestingly, there was no statistically significant difference in AP administration between patients with eHAT (13%) vs. those without eHAT (15.3%; *p* = 0.67).

As expected, patients who developed eHAT were more likely to require re-transplantation (52.2% vs. 6.2%; *p* < 0.01) than those without eHAT. Similarly, eHAT was associated with a lower graft survival at 1 year of 47.3% (95% CI: 39.9–54.7) compared to 87.9% (95% CI: 86.7–89.1) in those without eHAT, (*p* < 0.01). One-year patient survival was, however, not affected (82.4% vs. 90.5%, *p* = 0.07). The remainder of the patients with eHAT were treated with surgical revascularization (50%), endovascular therapy (5%) and prolonged anti-platelet therapy/anticoagulation (45%).

### Identifying Risk Factors for eHAT Including AP

In the total population (N = 836), we performed univariable Cox regression analysis to identify risk factors for eHAT. We found that recipient age (HR 0.97, 95% CI: 0.95–0.99) cryptogenic cirrhosis as the underlying liver disease (HR 2.85, 95% CI: 1.12–7.21), duration of surgery (HR 1.004; 95% CI: 1.001–1.007), intraoperative arterial thrombus formation (HR 5.21; 95% CI: 2.21–12.29), arterial conduit (HR 5.82; 95% CI: 2.09–16.23), hepatic artery reconstruction (HR 2.32; 95% CI: 1.22–4.41), arterial anastomosis redo (HR 5.64; 95% CI: 2.80–11.38), donor-recipient age difference greater than 15 years (HR 2.01; 95% CI: 1.09–3.68) and donor diabetes mellitus (HR 2.57; 95% CI: 1.01–6.52) were significantly associated with an increased risk of eHAT (Table 2). In contrast, AP was not associated with eHAT in the univariable analysis in the whole population (HR 0.80; 95% CI: 0.34–1.89), nor was the use of DCD grafts (HR 0.89, 95% CI: 0.47–1.70), nor DRI (HR 1.1, 95% CI: 0.55–2.19) nor graft steatosis (HR 1.06, 95% CI: 0.57–1.96). Next, we fitted multiple multivariable models (see methods), with the final model being selected by the lowest AIC (508.20) and highest AUROC (0.681). We found that the use of AP (aHR = 0.18; 95% CI: 0.05–0.59) was protective against eHAT while arterial redo (aHR = 4.33; 95% CI: 1.69–11.07), hepatic artery reconstruction (aHR = 3.72; 95% CI: 1.50–9.22), together with cryptogenic cirrhosis as the underlying liver disease (aHR = 4.25; 95% CI: 1.60–11.25) were consistently and independently associated with increased eHAT development (Table 3).

**TABLE 2:** Univariable Cox proportional hazards survival analysis of potential risk factors for eHAT in the overall population.

Variable	Univariable analysis		
	HR	95% CI	*p*-value
Age (years)	0.97	0.95–0.99	**0.02**
Cryptogenic cirrhosis	2.85	1.12–7.21	**0.03**
BMI (kg/m^2^)	0.99	0.94–1.06	0.92
MELD Score	1.005	0.97–1.04	0.77
Pre-LT TACE/TARE	0.63	0.22–1.76	0.38
Recipient Diabetes Mellitus	0.36	0.13–1.01	0.05
Type of graft			
DBD	1.003	0.55–1.82	0.99
DCD	0.89	0.47–1.70	0.72
Donor age (years)	0.99	0.97–1.01	0.27
Donor sex (male)	0.68	0.37–1.23	0.20
Donor BMI (kg/m^2^)	1.01	0.94–1.09	0.73
Donor 15 years older than recipient	2.01	1.09–3.68	**0.02**
Donor Diabetes Mellitus	2.57	1.01–6.52	**0.05**
Donor smoking	1.08	0.58–2.00	0.79
Donor Risk Index (DRI)	1.10	0.55–2.19	0.78
Donor steatosis (any degree)	1.06	0.57–1.96	0.85
Surgery duration (min)	1.004	1.001–1.007	**0.01**
Blood loss (L)	1.00	1.00–1.00	0.82
Fibrinogen use	0.55	0.31–1.03	0.06
Intraoperative arterial thrombus formation	5.21	2.21–12.29	**0.01**
Arterial conduit	5.82	2.09–16.23	**0.01**
Arterial redo	5.64	2.80–11.38	**0.01**
HA reconstruction	2.32	1.22–4.41	**0.01**
Peri-anastomotic bile leak^a^	2.48	0.77–8.01	0.12
Antiplatelet prophylaxis	0.80	0.34–1.89	0.62

Results are expressed as hazard ratio (HR) and 95% confidence interval (CI).

eHAT, early hepatic artery thrombosis; HR, hazard ratio; BMI, body mass index; MELD, Model for end-stage liver disease; DBD, donation after brain death; DCD, donation after cardiac death; HA, hepatic artery.

^a^ Bile leak before hepatic artery thrombosis.

The bold values indicate statistical significance.

**TABLE 3:** Final multivariable Cox proportional hazards survival model for risk factors for eHAT in the overall population.

Variable	aHR	95% CI	*p*-value
Age	0.98	0.95–1.02	0.46
Donor 15 years older than recipient	1.78	0.75–4.20	0.18
Cryptogenic liver cirrhosis	**4.25**	**1.61–11.25**	**0.01**
Surgery duration	1.002	0.99–1.005	0.39
Intraoperative arterial thrombus formation	1.90	0.57–6.25	0.29
Donor diabetes mellitus	1.80	0.63–5.15	0.27
Arterial conduit	1.43	0.40–5.15	0.57
Arterial anastomosis redo	**4.33**	**1.69–11.07**	**0.01**
Hepatic artery reconstruction	**3.72**	**1.50–9.22**	**0.01**
Antiplatelet prophylaxis	**0.18**	**0.05–0.59**	**0.01**

Results are expressed as adjusted hazard ratio (aHR) and 95% confidence interval (95% CI). This model had an AIC of 508.20 and an AUROC of 0681.

AP, antiplatelet prophylaxis; eHAT, early hepatic artery thrombosis.

The bold values indicate the variables with statistical significance.

### The Effect of Antiplatelet Prophylaxis in Patients With Risk Factors for eHAT

**FIGURE 1:**
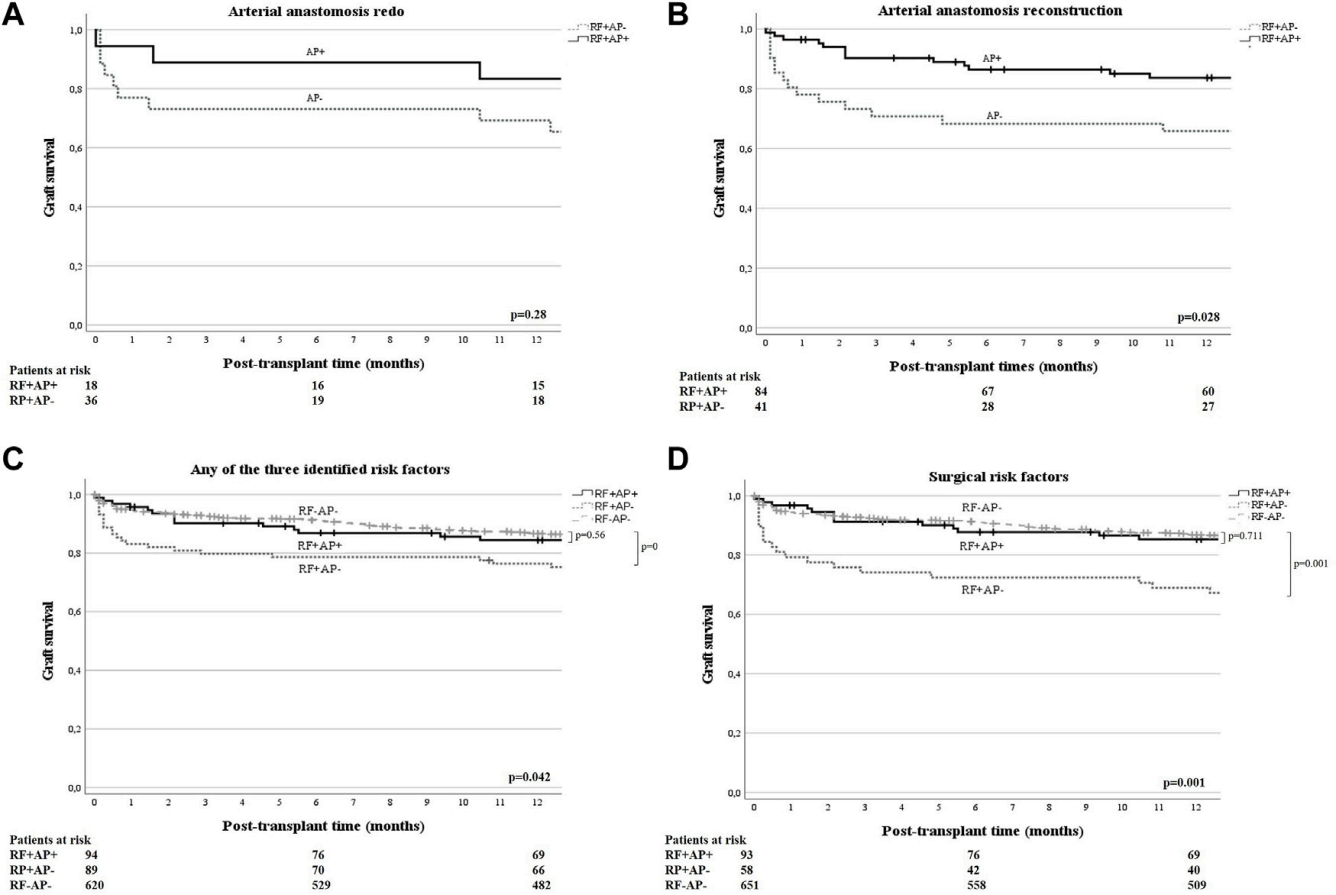
**(A, B)** One-year graft survival of patients with arterial redo [**(A)**, n = 44], or arterial reconstruction [**(B)**, n = 125], (i.e., independent eHAT RF derived from the multivariate model), stratified by use of antiplatelet prophylaxis (AP+/−) and compared by log-rank test. One year graft survival for RF+AP+ vs. RF+AP− was 83.3% (95% CI: 74.5–92.1) versus 69.2% (95% CI: 60.1–78.3) in patients with redo anastomosis [*p* = 0.28; **(A)**]; and 83.7% (95% CI: 79.5–87.9) versus 67.8% (95% CI: 60.4–75.2) in patients with arterial reconstruction [*p* = 0.03, **(B)**], respectively. **(C)** One year graft survival of patients with either cryptogenic liver cirrhosis and/or arterial reconstruction and/or anastomotic redo (i.e., anyRF+, n = 183), stratified according to the use of antiplatelet prophylaxis (AP+/−) and compared by log-rank test. An additional comparison was made with all other patients who did not have the identified risk factors and did not receive AP (surgRF−AP−, n = 620). One-year graft survival was 84.4% (95% CI: 80.6–88.2) for anyRF+AP+ versus 77.4% (95% CI: 70–81.8) for anyRF+AP− vs. 86.6% (95% CI: 85.2–88) *p* = 0.042. **(D)** One year graft survival of patients with either arterial reconstruction and/or anastomotic redo (i.e., surgRF+, n = 151), stratified by use of antiplatelet prophylaxis (AP+/−) and compared by log-rank test. An additional comparison was made with all other patients who did not have these surgical risk factors and did not receive AP (surgRF−AP−, n = 651). One year graft survival was as follows: in surgRF+AP+ 85.3% (95% CI: 81.5–89.1), in surgRF+AP− 70.4% (95% CI: 64.4–76.4), and in surgRF-AP 86.8% (95% CI: 85.5–88.1). Graft survival in patients with surgRF+AP+ was significantly better than in patients with RF+AP− (*p* = 0.018) and equal to all surgRF−AP− (*p* = 0.71).

Given that AP was not a significant predictor of eHAT in the univariable analysis of all (i.e., unselected) patients, but appeared to be a significant predictor in the multivariate model, we were interested in identifying in which population AP may be most beneficial. Therefore, we compared eHAT rates and survival outcomes between those who had identified risk factors and were given AP (RF+AP+) and those with RF not receiving AP (RF+AP−). First, in patients with cryptogenic cirrhosis (n = 35), only n = 2 patients received AP (i.e., ccRF+AP+) and n = 33 did not (ccRF+AP−). Although limited by low numbers, we found that the eHAT rates were not significantly different (0% vs. 15.2%, *p* = 0.55) and there was no significant difference in 1-year patient (*p* = 0.10) or graft survival (*p* = 0.19; Figure 1A) between those with and without AP. Second, in those with arterial anastomosis redo (n = 44), 18 received AP (redoRF+AP+) and 26 did not (redoRF+AP−). Here, the eHAT rate was significantly lower in redoRF+AP+ (5.6%) vs. redoRF+AP− (34.5%, *p* = 0.02). However, 1-year patient survival (*p* = 0.90) and graft survival (*p* = 0.28) were similar between the two groups (Figure 1B). Third, in patients who underwent arterial reconstruction (N = 125), 84 received AP (reconRF+AP+) and 41 (reconRF+AP−) did not. Again, the eHAT rate was significantly lower in the reconRF+AP+ group (3.5%) than in the reconRF+AP− group (24.4%; *p* < 0.01). Moreover, those with reconRF+AP+ had an improved 1-year graft survival of 83.7% (95% CI: 79.5–87.9) vs. 67.8%, (95% CI: 60.4–75.2; *p* = 0.03) in reconRF+AP (Figure 1C). Patient survival remained unchanged (*p* = 0.29).

Next, we evaluated the effect of AP in patients with at least one of the three risk factors (anyRF; n = 183) and found a significantly lower eHAT rate of 3.2% in anyRF+AP+ (n = 94) compared to the rate of 21.3% in anyRF+AP− (n = 89; *p* < 0.01) but no difference in 1-year patient (*p* = 0.96) or graft survival (*p* = 0.17) (Figure 1D). Following this observation, we then compared these two groups to the remaining patients in our cohort who did not have any of these three risk factors and who did not receive antiplatelet therapy (i.e., anyRF−AP−,n = 620), and found that those who had anyRF+AP− had a significantly worse graft survival (77.4% vs. 86.6%, *p* = 0.01), while graft survival in patients with anyRF+AP+ was similar to that in those without any RF (84.4% vs. 86.6%, *p* = 0.56). Finally, when evaluating the effect of AP in those with surgical RF only (i.e., either arterial redo or reconstruction, n = 151), the difference in eHAT rate became even greater with 3.2% in surgRF+AP+ (n = 93) versus 25.8% in surgRF+AP− (n = 58; *p* < 0.01). Moreover, surgRF+AP+ showed a 1-year graft survival of 85.3% (95% CI: 81.5–89.1) which was equivalent to the graft survival of 86.8% (95% CI: 85.5–88.1; *p* = 0.71) in patients who did not have any of the two surgical RF and no AP (surgRF−AP−, n = 651), whereas graft survival was significantly compromised in surgRF+AP-(70.4%; 95% CI: 64.4–76.4; *p* = 0.02) (Figure 1E). There was again no effect on 1-year patient survival (88.7% vs. 84.5% vs. 90.5%, respectively, *p* = 0.33).

## Discussion

In our study, which included 836 patients after liver transplantation, the eHAT rate was 5.5% and 15.2% received AP for surgical reasons. Although we did not find a significant association between the overall eHAT rate and the use of AP in the uncontrolled (univariable) analysis, AP was found to be independently associated with reduced eHAT rate (aHR = 0.18) in the multivariable model. In contrast, arterial anastomosis redo (aHR = 4.33), hepatic artery reconstruction (aHR = 3.72), and cryptogenic cirrhosis as the underlying liver disease (aHR = 4.25) were associated with an increased risk of eHAT. Interestingly, we showed that administration of AP in patients with any one of these risk factors significantly mitigated the risk of eHAT, especially in those who underwent either arterial redo and/or reconstruction. In this high-risk group, an 8-fold decrease in the rate of eHAT (3.2% vs. 21.3%) and an absolute difference in 1-year graft survival of 14.9% (85.3% vs. 70.4%) were seen in favor of AP. Indeed, after AP, graft survival in these high-risk patients became equivalent to that of patients without any of these eHAT risk factors. Therefore, our results suggest that AP may be recommended in all patients who underwent an arterial redo or reconstruction during transplant surgery.

While the real pathogenesis of eHAT remains unclear, it is typically attributed to a combination of donor, surgical, and recipient factors. Among the identified non-surgical RFs, we only found cryptogenic liver cirrhosis as an independent risk factor for eHAT. Although patients with so-called cryptogenic liver cirrhosis were labeled as such because no specific etiology could be identified at the time, we now know that in retrospect, a large proportion of this group of cryptogenic cirrhosis may have been suffering from metabolic dysfunction associated steatohepatitis MASH, since the typical clinicopathological features of MASH are known to fade once decompensated cirrhosis is established [14]. Indeed, among the patients in our cohort, 23% had DM and 20% had obesity. MASH, together with the other associated co-morbidities and systemic changes (systemic inflammatory milieu, intestinal dysbiosis, insulin resistance), may all contribute to a chronic inflammatory status that favors endothelial cell activation, lipid-derived oxidative injury, necroapoptosis, and ultimately, prothrombotic changes [15, 16]. So, while it is tempting to speculate that preceding MASH may have, at least in part, contributed to the increased risk of eHAT, we did not find a higher rate of eHAT in patients with confirmed MASH. Additionally, we could not identify a protective effect of AP due to the very small number of patients who received it (n = 2). Larger studies are needed to confirm these findings before firm conclusions can be drawn.

The most important RFs were, however, surgical in nature. The need to perform an arterial anastomosis redo during the transplantation surgery was found to be significantly associated with the development of eHAT, both in univariable and multivariable analysis. A redo is usually needed for technical issues such as anastomotic angulation or traction, or suboptimal arterial inflow resulting from spasm, intimal dissection or instant thrombus formation. Our results suggest that in this situation, the increased risk of HAT and graft failure can be mitigated by the administration of AP in the post-transplant setting. To the best of our knowledge, this factor has not been previously examined as a separate potential risk factor in other studies. Finally, in agreement with previous studies [3, 17], bench reconstruction of an anatomical variant or damaged hepatic artery also increased the risk of eHAT development, probably due to the increased number of arterial anastomoses combined with an abnormal morphology compared to the standard end-to-end/single arterial anastomosis technique [18, 19].

The most important finding in this study was the protective effect of AP on the rate of eHAT in high-risk patients, while this did not appear to be the case in the overall population. Although AP was mainly used for a variety of surgical difficulties during arterial anastomosis, not all patients with these difficulties actually received AP. This may have increased the number of eHAT in the group without AP, rendering it not beneficial in the overall population. Our findings are consistent with recent publications. Wolf et al. assessed the use of AP in 354 consecutive, and thus unselected, LT recipients and, like us, did not identify any benefit [13]. However, a more recent study found that prophylaxis with 325 mg/day of aspirin initiated immediately after surgery and continued for 3 months in 439 unselected patients led to a decreased eHAT incidence from 3.6% to 0%, without increasing the risk of bleeding [20]. However, such high dosing may come at the expense of other adverse events such as peptic ulcerations and liver/kidney toxicity and is probably not to be recommended in all post-LT patients.

On the other hand, in selected high-risk patients, AP was shown to be very beneficial. Indeed, when we selected patients who had at least one of the independent risk factors for eHAT, AP was associated with an 8-fold decreased rate of eHAT (3.2% vs. 25.8%) compared to those with the same RFs who did not receive AP. Also, 1-year graft survival was significantly improved in the AP group while risks of bleeding were similar. Our study is in line with another retrospective single-center study that found an 82% relative risk reduction in high-risk patients (defined as those who received grafts from donors after a cerebrovascular accident and/or use of an iliac conduit at transplantation), without any recorded bleeding episodes during follow-up [17]. Our results therefore confirm that AP should be reserved for these selected high-risk patients.

Two other previously described surgical risk factors for eHAT (i.e., the use of arterial conduit and intraoperative arterial thrombosis) [21, 22], were identified as potential risk factors for eHAT in our univariable analysis, but failed to remain independent risk factors in the multivariable analyses. This may be due to the small number of patients in each group (n = 15 and n = 27, respectively). However, in retrospect, we observed that 33% and 48% of these patients, respectively, received AP, and none (0%) of the patients who received AP developed eHAT compared to 40% in those with an arterial conduit and 42.9% in those with intraoperative arterial thrombosis who did not receive AP. Although not a direct result of our study, it is reasonable to assume that AP may be protective in these situations as well, something that could be further explored in larger datasets with more events.

Moreover, the use of antiplatelets may also have long-term additional protective effects on these patients in terms of preventing cardiovascular events [6] and even reducing the incidence of acute rejection episodes as suggested by the study of Oberkofler et al [12].

Our study has several strengths and limitations that need to be addressed. Strengths of this study include a relatively large and uniform dataset with complete follow-up and comprehensive data collection on a large subset of potentially important recipient, donor and surgical RFs. Despite this, our study was limited by the fact that the event rate was still low, resulting in limited power and potential overfitting in the case of multivariable analysis as many potential risk factors were identified from the univariable analyses. We tried to overcome this by fitting multiple models and using the AIC and AUROC to select the best model fit. Second, due to the retrospective nature of this study, there are missing data that could have underestimated the role of some potential RFs. Third, we could not completely retrieve the individual reasons why some patients with potential surgical RFs were not prescribed AP, which could have introduced potential confounding by indication. Although intraoperative blood loss was higher in those not receiving AP (indicating fear of postoperative bleeding as a possible reason) none of the post-LT coagulation parameters indicated worse coagulation or potentially higher risk of bleeding in these patients. Finally, the observational and retrospective, rather than interventional, nature of our study does not allow us to draw definite conclusions about the beneficial effects of AP and larger, prospective studies may be needed to confirm our findings.

## Conclusion

Patients who underwent arterial redo or hepatic artery reconstruction, or who had cryptogenic liver cirrhosis as an indication for LT have an increased risk of eHAT. In selected high-risk patients, AP was associated with an 8-fold reduced risk of eHAT and significantly improved graft survival. Our results warrant increased vigilance for eHAT in the presence of these RFs and suggest a possible protective role of antiplatelet prophylaxis in these selected cases.

## Data Availability

The raw data supporting the conclusions of this article will be made available by the authors, without undue reservation.
